# Synovial Plica Syndrome of the Knee: A Commonly Overlooked Cause of Anterior Knee Pain

**DOI:** 10.1055/s-0037-1598047

**Published:** 2017-02-15

**Authors:** Paul Yuh Feng Lee, Amy Nixion, Amit Chandratreya, Judith M. Murray

**Affiliations:** 1 South Wales Orthopaedic Research Network, WelshBone, Cardiff, Wales, United Kingdom; 2 Department of Orthopaedic Surgery, ABMU LHB, Princess of Wales Hospital, Bridgend, Wales, United Kingdom; 3 Department of Orthopaedic Surgery, Royal Glamorgan Hospital, Llantrisant, Wales, United Kingdom

**Keywords:** plica, knee, synovial shelf syndrome, anterior knee pain

## Abstract

Synovial plica syndrome (SPS) occurs in the knee, when an otherwise normal structure becomes a source of pain due to injury or overuse. Patients may present to general practitioners, physiotherapists, or surgeons with anterior knee pain with or without mechanical symptoms, and the diagnosis can sometimes be difficult. Several studies have examined the epidemiology, diagnosis, and treatment of SPS. We review these resources to provide an evidence-based guide to the diagnosis and treatment of SPS of the knee.

## What is SPS of the Knee and Who Gets it?

**Fig. 1:**
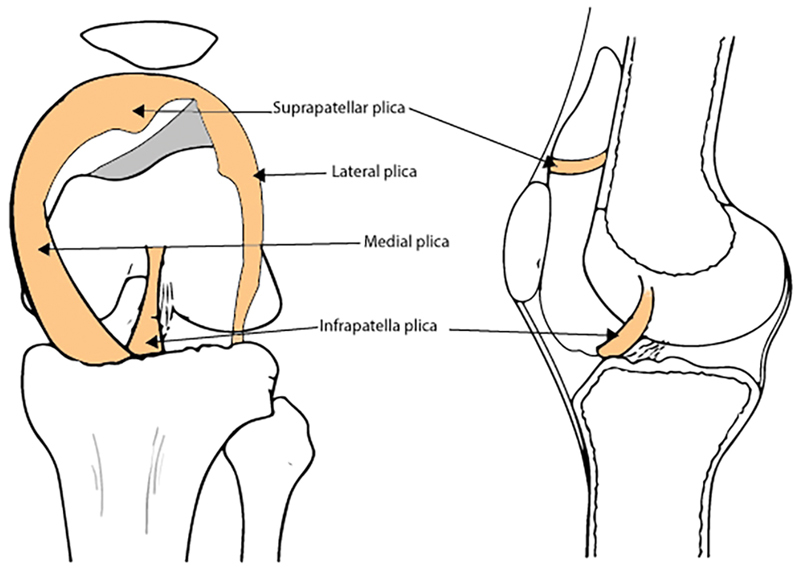
Location of the synovial plica inside the knee joint.

SPS encompasses a collection of symptoms, usually affecting people of both sexes in their first through third decade. 1 2 3 Patients often mention anterior knee pain, clicking, clunking, and a popping sensation on patellofemoral loading activity such as squatting. 2 3 4 5 6 7 8 There is wide variation in reported prevalence of SPS, ranging from 3 to 30% in European population studies; most studies cite a figure of approximately 10%. 2 3 6 9 10 11 According to their location, the synovial plicae are classified as suprapatellar, mediopatellar, infrapatellar, or lateral ( Fig. 1 ); the medial plica is the most commonly symptomatic one. 2 3 7 9

## Anatomy and Pathology

Plicae are inward folds of the synovial lining and are present in most knees. 2 3 7 In their normal state, they are thin and pliable and appear almost transparent. Proximally, the mediopatellar, which is most important clinically, is attached to the articularis genus muscle, while it runs distally to the intra-articular synovial lining and blends into the medial patellotibial ligament on the medial aspect of the retropatellar fat pad. 12 13 Depending on its position, size, and elasticity, the plica may impinge between the quadriceps tendon and femoral trochlea at 70 to 100 degrees of knee flexion, causing mechanical symptoms. 3 14 In some individuals, the size and elasticity of the synovial fold can be more developed compared with others. Plicae become pathological when its inherent qualities change due to an inflammatory process that alters the pliability of synovial tissue. A pathological synovial plica that has been through this inflammatory process can become inelastic, tight, thickened, fibrotic, and sometimes hyalinized. A synovial plica affected by such changes may bowstring across the femoral trochlea, causing impingement between the patella and femur in knee flexion. 2 8 15 16 A pathological synovial plica can express a plethora of symptoms; the clinical history usually discloses nonspecific anterior or anteromedial knee pain, which has led to the conception of the term *synovial plica syndrome* . 1 5 6

## Embryology

There are debates about the formation of the knee joint during intrauterine fetal life. The most widely accepted theory is that in the eighth week of fetal life, mesenchymal condensations representing cruciate ligaments and menisci emerge, and a proper joint cavity separating the three cartilaginous anlagen can be identified. 17 The synovial septa are partially resorbed over the next 2 weeks, and the patellofemoral, femoromeniscal, and meniscotibial regions amalgamate to create a larger cavitation which becomes the knee joint. 2 In areas where the mesenchymal cavitation fails to coalesce, persistent mesenchymal tissue may differentiate into folds of synovium, and if large enough, are considered as plicae. They are frequently present bilaterally.

## Symptoms

Most cases of knee SPS are idiopathic, and symptoms have been estimated to be bilateral in up to 60% of cases, although they may not manifest concurrently. 9 Other causes or associations have been identified associated with trauma, overuse injuries, hematoma, diabetes, and inflammatory arthropathy. In adolescence, symptoms can occur during a period of growth spurt. Any primary disorder of the knee capable of producing transient or chronic synovitis may therefore be implicated in the development of a pathological plica. Due to the embryological theory behind this condition, there is some evidence to suggest a genetic component for knee SPS, although the exact basis of this has not yet been established. 2 8 15 18

Patients may report aggravation of symptoms with overuse or heavy activities involving flexion and extension of the knee. The synovial plica is directly implicated as part of the knee joint and is indirectly attached to the quadriceps musculature as the position of the plica is dynamically controlled during knee flexion and extension because of its attachment to the fat pad. Plical irritation is more common in patients who have poor quadriceps tone or any significant muscle imbalance around the knee. 12 It has been reported that prolonged flexion of the knee can initiate pain, which can be the situation when sleeping at night, and can sometimes it be troublesome for patients. 19 Deterioration of symptoms is not the obligatory clinical course of the condition, but how to identify those patients who will experience progressively worsening symptoms without treatment has not been satisfactorily resolved.

## How Is It Diagnosed?

One of the most important points in diagnosing knee SPS is obtaining an appropriate history from the patient. It is a clinical diagnosis that may be supported by specific tests and imaging. The diagnosis should be suspected in patients of any age, although it is less common among young children below the age of 10 years, in whom the diagnosis is less likely to be idiopathic and often associated with meniscal pathology. 20 21 Some patients will report a history of blunt trauma or twisting injury with subsequent development of an effusion. After an initial injury has settled, they may continue to experience pain localized at the medial plica associated with fibrosis. 19

Patients may give a history of anterior knee pain following strenuous physical work or athletic activity, which requires repetitive flexion and extension motion of the knee, which irritates the patellofemoral joint. 17 These activities may include ascending and descending stairs, squatting, bending, or arising from a chair after sitting for an extended period of time. In addition, they may note difficulty with sitting still for long periods of time without having to move and stretch their knees. Anterior knee pain, although not a condition in its own right, frequently receives unjustified status of a diagnosis. It is the cardinal symptom of a pathological synovial plica and present in almost all patients who carry the condition. 2 22

Symptoms are classically absent during the initial phases of sporting activities but occur with a delay of a few months and often force the individual to discontinue. Due to the mechanical nature of the disease, intensified activity will increase the degree of plica irritation and may explain the prevalence of the condition in sporting individuals.

Patients commonly report intermittent nonspecific anterior knee pain, snapping, clicking, catching, clunking, grinding, “giving way,” or a popping sensation along the inside of the knee during flexion and extension. The knee may be tender to the touch, swollen, and stiff ( Table 1 ). Symptoms are often clinically indistinguishable from other intra-articular conditions such as meniscal tears, articular cartilage injuries, or osteochondritic lesions, creating a diagnostic conundrum. 2 This clinical ambiguity together with a lack of awareness often leads to a failure to diagnose and treat this condition. 23 Patients may also complain of atypical symptoms including pseudolocking of the knee or may even manifest as vaguely localized pain in the anterior shin, sometimes radiating proximally to the groin. Where symptoms are isolated to locking and sharp pain, meniscal pathology should first be ruled out, as a diagnosis of knee SPS is less likely. Osteoarthritis of the knee is not a bar to investigation or treatment for SPS, although patients should be counseled that they may experience incomplete relief of symptoms or no improvement at all, depending on the extent of osteoarthritic involvement.

**Table 1:** Symptoms and signs of knee synovial plica syndrome

• Anterior knee pain (parapatella)
• Snapping sensation along the inside of the knee as the knee is bent
• Clicking, catching, clunking, grinding, popping
• Tender to the touch
• Felt as a tender band underneath the skin ( Fig. 1 )
• Knee effusion, swelling
• Pain on squatting
• Locking, stiffness, giving way

## Clinical Examination

**Fig. 2:**
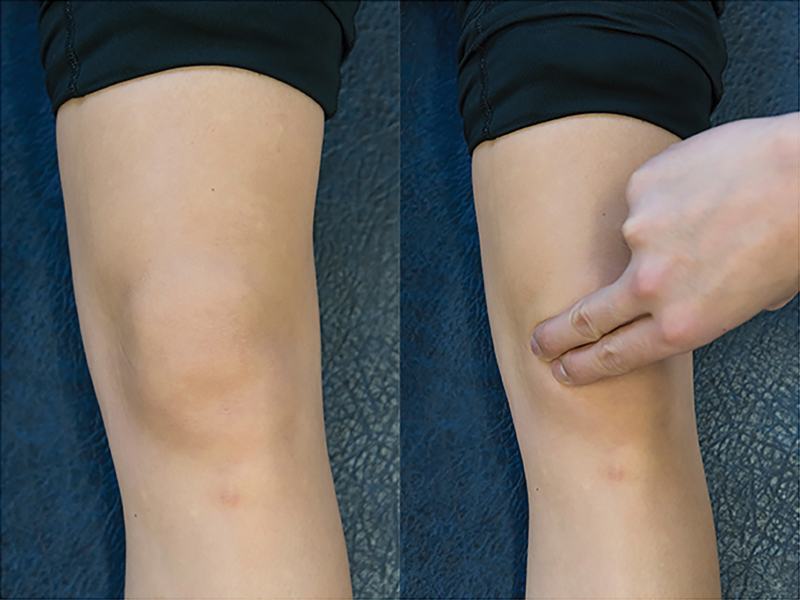
Media plica palpation. Patient positioning: supine with the knee extended and relaxed. The examiner stands on the affected side. Action: the examiner palpates patient's anterior compartment of the knee, starting from inferior medial region. Positive finding: pain and/or cordlike structure in the knee is indicative of a plica.

When examining the knee for abnormal plicae, it is important to make sure that the patient is relaxed, which is usually accomplished by having the patient lie supine on the examining table with both legs supported. The examiner can then palpate for the plica by rolling one finger over the plica fold, which is located around the joint lines in anterior knee compartment ( Fig. 2 ). A palpable plica will present as a ribbonlike fold of tissue, which can be rolled directly against the underlying medial femoral condyle. While some patients may have a sensation of mild pain when palpating the synovial plica, it is important to ascertain while performing this test if this reproduces their symptoms. 12 23 24 25 It is also very important to compare the sensation to the opposite knee to see if there is a difference in the amount of pain produced.

As with any other physical diagnosis, it is important to concurrently ascertain if there are other possible areas of pathology in structures that are located close to the synovial plica. In acute injuries, other common soft tissue problems such as meniscal, patellofemoral, and cruciate and collateral ligament injuries should be ruled out before narrowing the diagnosis to a synovial plica. 12

**Fig. 3:**
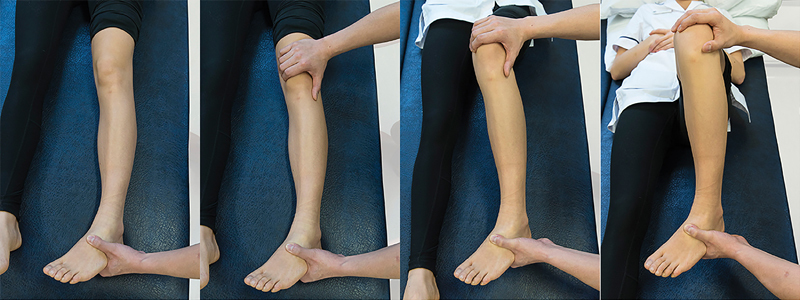
Hughston's plica test. Patient positioning: supine with the knee fully extended and relaxed. The examiner stands on the affected side, placing one hand around the heel and the palm of the other hand over the lateral border of the patella with the fingers over the medial femoral condyle. Action: the examiner flexes and extends the patient's knee while internally rotating the tibia and pushing the patella medially. Positive finding: pain and/or popping in the knee is indicative of an abnormal plica. It is typically in the range of 30 to 60 degrees toward extension.

**Fig. 4:**
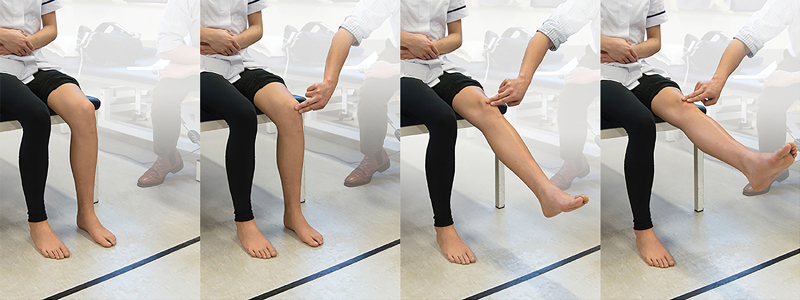
Stutter test. Patient positioning: sitting on the side of the bed with knee flex to 90 degrees. The examiner crouches down to knee level placing the index and middle fingers on the center of the patella. Action: the examiner asks the patient to extend the knee slowly while keeping the fingers on the patella and watches its movement. Positive finding: if the patella stutters or jumps during the course of movement, it is indicative of a plica. It is typically in the range of 45 to 70 degrees toward extension. Crepitus of the patella may also be felt.

In more severe disease, examination may identify a cordlike structure in the anteromedial compartment of the knee with palpation ( Fig. 2 ), often producing clicking on knee extension ( Video 1 ). The Hughston's plica test ( Fig. 3 ) and Stutter test ( Fig. 4 ) are provocative tests commonly used to support a diagnosis of SPS. 12 These tests are considered to be more supportive of the diagnosis when both tests are positive, but are less reliable when used individually, with wide variation in their reported sensitivity and specificity. 5 7 8 20 21 24

**Video 1** Visual appearance of the medial plica during inspection. Online content including video sequences viewable at: www.thieme-connect.com/products/ejournals/html/10.1055/s-0037-1598047.

## How Is It Investigated?

Once a clinical diagnosis of SPS has been implicated, clinicians should attempt to identify the specific cause for the symptoms. Any reversible or transient causes may contribute to an improvement or resolution of the symptoms. Secondary, less common causes of SPS include rheumatoid arthritis, bleeding into the knee associated with hemophilia, and space-occupying lesions within the knee. Although rheumatoid arthritis, diabetes, and hemophilia are thought to be associated with SPS, it is neither useful nor cost-effective to screen patients presenting with SPS symptoms with blood or glucose testing in the absence of other suggestive history or clinical evidence of disease.

It is important to rule out patella subluxation, osteochondritis dissecans, pathological fractures, chondromalacia, or other bony pathology, which could be contributing to the irritation of the synovial plica. There has been a report suggesting that the synovium in the knee is 15 times more sensitive compared with any other joint tissue and thus irritation of the synovium can lead to significant symptoms. 19 Although the existence of chondromalacia patellae is not often an important contributing factor to anterior knee pain, it may case mild synovial irritation. It is therefore recommended to obtain a weight bearing anteroposterior, lateral, and patella skyline radiographs. Many patients who have SPS have normal radiographs.

Arthrography, ultrasound, and magnetic resonance imaging (MRI) can all demonstrate the presence of a plica; however, they are unreliable in predicting which plicae are pathological. 6 26 Although these imaging techniques are useful, we suggest that they are not required to make an initial clinical diagnosis of SPS or to initiate management in the primary care environment. Their use is better suited to assessment in a specialist environment and for evaluation of complex cases, relapse of symptoms, and for assessing patient suitability for surgery.

The combination of the history, examination, and results of these specific tests will give clinicians a good, albeit subjective, impression of the likelihood of SPS of the knee as a clinical diagnosis. SPS is a likely diagnosis given its considerable prevalence; however, the condition can be confused with other diseases ( Table 2 ). These should be considered as alternative or coexisting diagnoses, depending on the presentation and age of the patient.

**Table 2:** Differential diagnoses for knee synovial plica syndrome

• OCD
• Meniscus pathology
• Patella femoral subluxation
• Hoffa fat pad syndrome
• Osteoarthritis of the knee; this can often coexist with SPS
• Chondromalacia
• Inflammatory arthropathy of the knee; this may coexist with SPS (look for features of systemic disease as well as joint-specific symptoms)
• Snapping ITB syndrome; this may coexist with SPS, usually on the lateral side
• Ligament instability, such as ACL, MCL
• Chondromatosis
• PVNS
• Patella tendinosis

Abbreviations: ACL, anterior cruciate ligament; ITB, iliotibial band; MCL, medial collateral ligament; OCD, osteochondritis dissecans; PVNS, pigmented villonodular synovitis; SPS, synovial plica syndrome.

## How Is It Managed?

Providing any other treatable diagnoses responsible for anterior knee pain have been excluded and the diagnosis is confident, there are several treatment options available for SPS. Management of SPS has been discussed in the literature over the past few decades; however, the supporting evidence is of variable availability and quality.

## Conservative Management

Conservative treatment can be managed in primary care and referral made for orthopaedic opinion, if required ( Table 3 ). Studies focused exclusively on treating SPS are fairly limited (most relating to “patellofemoral pain syndrome” or “anterior knee pain”). While it is an independent pathology and should be differentiated from other causes of anterior knee pain or pain syndrome, there is evidence to suggest that SPS responds well to general conservative measures such as activity modification, exercise therapy, and symptomatic treatment. 5 12 Conversely, some authors have published findings that do not support conservative management as a particularly successful solution, 2 which may be due a variety of factors, but this evidence has generally been low-level and based upon small, retrospective cohorts. Most would consider it best practice to initially treat suspected SPS of the knee conservatively, after ruling out any other conditions that would warrant other invention. Treatment is usually focused on reducing exacerbating factors, allowing irritation and inflammation to subside before building limb, and core strength should be sufficient for many patients, although clinical results from this regime have not been statistically analyzed.

**Table 3:** When should I refer?

• Diagnostic uncertainty
• Failed conservative treatments
• Severe symptoms or noticeable functional limitation
• Relapse after successful physiotherapy
• Recurrence after arthroscopic plica resection surgery
• Request by patient

**Activity Modification, Analgesia, Exercise Therapy**

A recent Cochrane review of 31 studies surrounding anterior knee pain, involving 1,690 participants, found a volume of low quality yet consistent evidence supporting the use of exercise therapy as treatment. It was suggested that use of exercise therapy alone resulted in a clinically important reduction in symptoms, increase in function, and improved overall recovery. 20 Considerable variance between studies in results, patient populations and diagnosis was noted, but it was felt that no harm was likely to be caused by using exercise therapy to treat anterior knee pain of various etiologies, and benefits were often clearly demonstrated. The best form of exercise therapy or ideal duration was not clarified, and success often depended on severity of symptoms. Resulting from these uncertainties, it was recommended that definitive agreed research questions should be investigated, using standardized diagnostic criteria and outcome measurements on a multicenter basis.

**Fig. 5:**
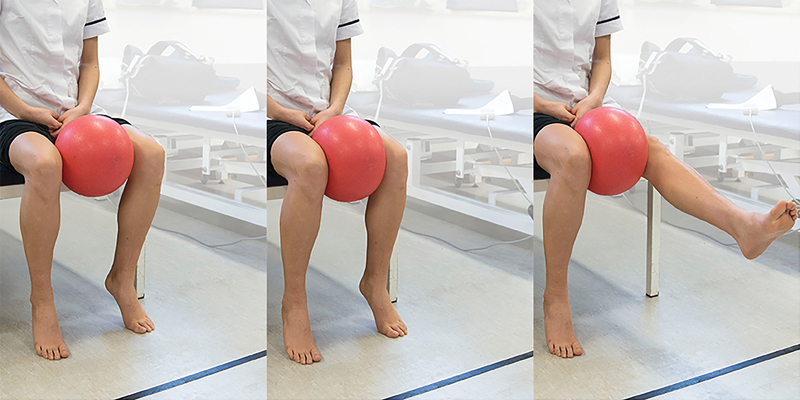
Vastus medialis oblique exercise. Patient positioning: sitting on the side of the bed or chair, with a ball (football) placed between the knees. Action: adduction force applied to knee to hold the ball between the legs, extend and flex the knee slowly (over 5 seconds), and focus on controlling the medial side of the quadriceps muscle.

Patients can initially be advised to modify their activities that are known to exacerbate plica syndrome (high-impact loading such as jumping, squatting, or lunging). Topical or oral nonsteroidal anti-inflammatories and paracetamol are likely to be sufficient in relieving pain. Rest, ice, and elevation may provide additional relief during acute episodes. When symptoms allow, an exercise program that aims to strengthen the vastus medialis oblique muscle (often weak in patients with anterior knee pain) along with the general quadriceps compartment ( Fig. 5 ) and lessen the antagonistic effect of the flexor muscles by stretching can be commenced. 12 27 This should be planned and initially undertaken with the supervision of a physiotherapist or sports medicine specialist, who has a thorough understanding of the biomechanical structure of the knee and the factors that influence its function.

**Fig. 6:**
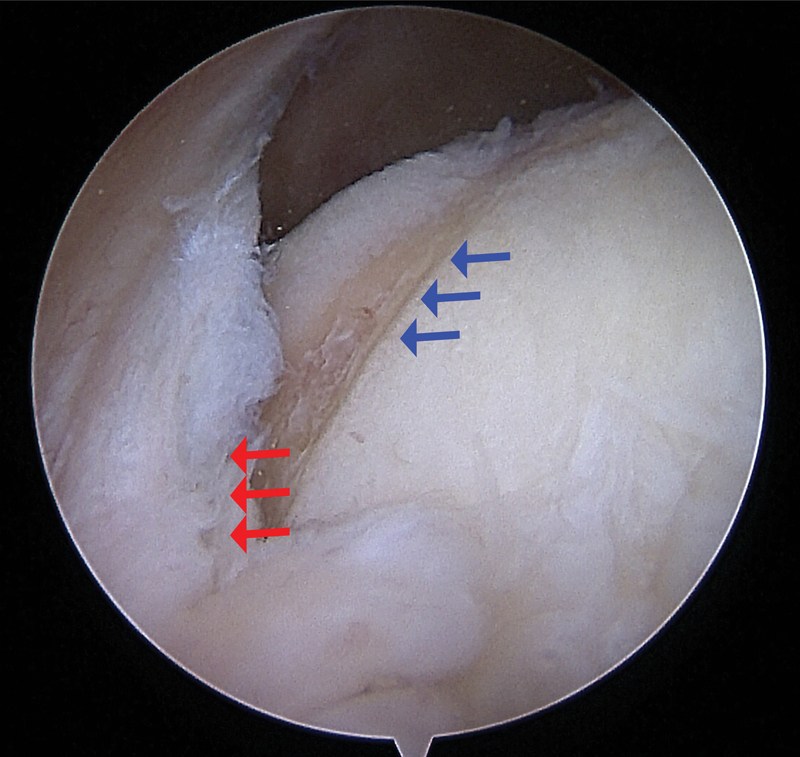
Arthroscopic photo showing the cartilage surface of the medial femoral compartment being eroded by the synovial plica. Red arrows indicated the plica and blue arrows demonstrate the erosion of the chondral surface of the femur.

## Taping

Despite historical use, anecdotal support, and patient favor, there is insufficient evidence to justify long- or short-term use of patellar taping in the treatment of anterior knee pain, and no clinical benefit has been attributed to its use. 21 28 The effects of taping around the patella have been shown on MRI assessment to have statistically improved tracking, 29 and clinical results have been reported to have good short-term outcomes, 2 with patients often reporting instantaneous results, but a recent systematic review surrounding the practice did not support the use of taping as a long-term treatment or even as part of a short-term intervention. 28 There are emerging studies on the use of stabilizing knee braces for anterior knee pain, but there is currently no evidence to suggest any benefit in SPS. As many braces compress the anterior compartment of the knee, it may even exacerbate symptoms.

## Intra-articular Injection

The use of intra-articular corticosteroid injection can be a useful adjunct (especially in the early stages before pathological change is established) and has been shown to reduce pain to a level that allows the patient to engage in physical therapy. 2 There is no evidence to support it as a stand-alone treatment and is unlikely to address the underlying cause of plica irritation. Intra-articular anesthetic injection with 1% lidocaine can help differentiate between true intra-articular and extra-articular pathology. Due to the technical difficulty, injection directly into the thin plica band is not recommended because reliable placement of the needle during the injection is impossible without image guidance. 30 Although knee arthroscopy remains the most definitive method for diagnosis of SPS, it is generally not recommended as a diagnostic test.

## Surgical Management

Patients who do not experience a significant improvement despite compliance with conservative measures over a reasonable period of time (3–6 months) should be offered further investigation and management ( Table 4 ). While SPS is primarily a clinical diagnosis, arthroscopy of the affected joint is the best dynamic way to confirm diagnosis ( Fig. 6 and Video 2 ) and the absence of other pathologies in a knee that is persistently painful. 24 It used to be fairly common practice to excise any identifiable plica during investigative arthroscopy, even if the presentation of pain did not necessarily correspond with the presence of the plica, or other pathologies were found. As synovial tissue is highly sensitive and has a propensity to fibrose if incompletely resected, many of these patients may have had suboptimal results and continuing symptoms if the true cause was not addressed and healthy synovial tissue disrupted. 24 26 The plica should only be resected if the diagnosis is clearly supported by examination and history, and conservative measures have been unsuccessful as supported by a cohort of 80 patients with arthroscopies in which 70 with confirmed plicae demonstrated a high level of immediate relief. 24 At arthroscopy, the plica should also be pathological in appearance or clearly causing significant impingement on movement, with no evidence of any other intra-articular pathology that correlates with symptoms. 2 12 24

On the rare occasions that surgical treatment is warranted, outcomes have been documented as good. Studies suggesting that approximately 10% of patients will continue to have disabling problems and 26% will have occasional problems but will be able to return to activity, with the large majority being symptom-free and able to return to previous level of activity. 2

## Conclusion

SPS of the knee is common and is seen in both community and hospital practice. A diagnosis of SPS should be suspected in patients with intermittent pain, swelling, and snapping sensation affecting the knees, which is associated with activity that involves increased loading of the patellofemoral joint.

Nonoperative strategies are usually successful for early or mild disease, or where advanced disease is associated with minimal symptoms. Conservative management requires good compliance from the patient, but is often sufficient in reducing symptoms, which will not necessarily be recurrent. Further research is required to give better evidence for a more definitive nonoperative treatment regime. Where nonoperative strategies fail, synovial plica resection provides good results and high levels of reported satisfaction for most patients.

## Sources and Selection Criteria

We researched PubMed, the Cochrane Library, and the Cumulative Index to Nursing and Allied Health Literature to identify source material for this review.

We examined available evidence published in the English language for the diagnosis and treatment of SPS, specific to the knee joint. The search terms used were “synovial shelf” “plica,” “plica syndrome,” “knee plica syndrome,” “synovial plica,” “knee synovial plica,” and “knee synovial syndrome.” There were no well-conducted large randomized trials and therefore “anterior knee pain” was also included. We selected and examined smaller randomized trials as well as case series, cohort studies, and observational reports where these provided the only evidence.

**Table 4:** Primary and secondary approach to knee synovial plica syndrome

**The primary care approach**
If the symptoms are related to the patellofemoral joint and there is no true locking or giving way with normal radiology, a nonoperative regime can be commenced. Activity modification should be advised; low impact exercise, weight reduction (if appropriate), and supportive foot wear may all be of benefit. 20 21 As SPS is not necessarily progressive, simply modifying activity and lifestyle for a period may sufficiently reduce symptoms. If no better after a minimum of 3 months of conservative treatment, when activity level cannot be indefinitely altered, and in the absence of other intra-articular pathology, referral to orthopaedics in a secondary care setting is recommended. 2 20
**Secondary care approach**
Arthroscopic examination is the most reliable method for diagnosis of SPS, and plica resection offers a mechanical solution and has good clinical results in selected complex and resilient cases ( Fig. 6 and Video 2 ). 31 However, it carries the burden of common surgical intervention and can also cause further irritation and scarring of the synovial plica.

Abbreviation: SPS, synovial plica syndrome.

**Video 2** Arthroscopic video of plica demonstrating impingement of the femur. Online content including video sequences viewable at: www.thieme-connect.com/products/ejournals/html/10.1055/s-0037-1598047.
